# Lung development and immune status under chronic LPS exposure in rat pups with and without CD26/DPP4 deficiency

**DOI:** 10.1007/s00441-021-03522-8

**Published:** 2021-10-04

**Authors:** Andreas Schmiedl, Inga Wagener, Meike Jungen, Stephan von Hörsten, Michael Stephan

**Affiliations:** 1 Functional and Applied Anatomy, Hannover Medical School, Carl-Neuberg Str. 1, 30625 Hannover, Germany; 2 Department of Experimental Therapy University Hospital Erlangen and Preclinical Experimental Center (PETZ), Friedrich-Alexander-University Erlangen-Nürnberg, Bavaria, Germany; 3 Clinic for Psychosomatics and Psychotherapy, Hannover Medical School, 30625 Hannover, Germany; 4 Biomedical Research in Endstage and Obstructive Lung Disease Hannover (BREATH), German Center for Lung Research (DZL), 30625 Hannover, Germany

**Keywords:** Rat, Lung development, Lipopolysaccharide (LPS), CD26/DPP4, Immune cells

## Abstract

Dipeptidyl-peptidase IV (CD26), a multifactorial integral type II protein, is expressed in the lungs during development and is involved in inflammation processes. We tested whether daily LPS administration influences the CD26-dependent retardation in morphological lung development and induces alterations in the immune status. Newborn Fischer rats with and without CD26 deficiency were nebulized with 1 µg LPS/2 ml NaCl for 10 min from days postpartum (dpp) 3 to 9. We used stereological methods and fluorescence activated cell sorting (FACS) to determine morphological lung maturation and alterations in the pulmonary leukocyte content on dpp 7, 10, and 14. Daily LPS application did not change the lung volume but resulted in a significant retardation of alveolarization in both substrains proved by significantly lower values of septal surface and volume as well as higher mean free distances in airspaces. Looking at the immune status after LPS exposure compared to controls, a significantly higher percentage of B lymphocytes and decrease of CD4^+^CD25^+^ T cells were found in both subtypes, on dpp7 a significantly higher percentage of CD4 T^+^ cells in CD26^+^ pups, and a significantly higher percentage of monocytes in CD26^−^ pups. The percentage of T cells was significantly higher in the CD26-deficient group on each dpp. Thus, daily postnatal exposition to low doses of LPS for 1 week resulted in a delay in formation of secondary septa, which remained up to dpp 14 in CD26^−^ pups. The retardation was accompanied by moderate parenchymal inflammation and CD26-dependent changes in the pulmonary immune cell composition.

## Introduction

CD26/DPP4, called CD26 in the following, is mainly expressed in kidneys and lungs and is involved in different inflammation processes (Mentlein 1999; Klemann et al. 2016). Adult asthma-induced CD26/DPP4-deficient rats exhibited an attenuated inflammation combined with a reduced influx of regulatory T cells and a reduced upregulation of surfactant proteins (Schmiedl et al. 2010). Also, a reduced inflammation with differential expression of surfactant proteins in CD26/DPP4 deficient rats was seen after lipopolysaccharide (LPS) administration (Zientara et al. 2019). LPS is the biologically active component and primary recognition element in the wall of gram-negative bacteria (Rossol et al. 2011). Therefore, LPS was frequently used to induce lung inflammation in adult animals (Kawasaki et al. 2018; Liu et al. 2013), newborns (Franco et al. 2002), or fetuses (Cao et al. 2009; Kramer et al. 2009; Ueda et al. 2006). The activated signal cascade leads then to an expression of immunoregulatory genes associated with synthesis and release of pro- and anti-inflammatory cytokines like IL-1, Il-6, Il-8, and Il-10 and tumor necrosis factor α (Martin and Frevert 2005; Takeda et al. 2003).

Stages of lung development in mammals are similar (Zoetis and Hurtt 2003). However, rodents are born with morphologically immature lungs comparable to human premature infant (Burri 2006). Alveolarization starts on postnatal day (day(s) postpartum, dpp) 4 in rats. So, the developmental lung stages dpp 3–5 are comparable with those of infants, which are born earlier than the 30th week postmenstrual fetal stage. Furthermore, this time period is a critical window, because exposure to injurious noxa can disrupt lung alveolarization and vascularization (Nardiello et al. 2017). After bulk alveolarization combined with vascular maturation lasted up to dpp 21, continued alveolarization occurred until adulthood (Schittny 2017). We have already shown that CD26 deficiency delayed the postnatal lung development (Hupa et al. 2014; Wagener et al. 2020) and that high doses of postnatal LPS inhalation (100 µg/2 mL NaCl) given for 10 min 1 day before and 1 day after the onset of alveolarization produced an acute but weakly pronounced inflammation and a delay in morphological lung development with a reduced morphological recovery after the end of LPS exposure in dependence of CD26 deficiency (Wagener et al. 2020). While this procedure resembles an acute injury, the repetitive administration of LPS over 1 week used in the present is more comparable with a chronic inflammatory disorder (Wang et al. 2009), which is a possible precondition to develop bronchopulmonary dysplasia (BPD) (Baker and Alvira 2014). BPD is a chronic disorder of the developing lung that occurs in preterm infants secondarily to an imbalance between lung injury and repair (Jobe 2015), often leading to an impairment of alveolarization (Coalson 2006). Several authors propose that postnatal infection /inflammation is a more important precursor for the development of BPD than antenatal infection (Jensen and Schmidt 2014; Ballard et al. 2016). Furthermore, inflammation caused by LPS is necessary to eliminate the pathogen. Therefore, an adequate immune response is a precondition (Hotchkiss et al. 2013). However, chronic exposure to low-dosed endotoxins such as LPS in early life may protect against atopic sensitization and IG-E-mediated diseases, but may negatively affect the lung accompanying wheezing according to the so-called hygiene hypothesis (Eder and von Mutius 2004). Furthermore, chronic exposure to endotoxin may, in contrast to acute LPS exposure, lead to some tolerance of endotoxins, leading to weaker LPS impact, e.g., after intraamniotic infection as shown by others (Kramer et al. 2009; Kallapur et al. 2007; Collins et al. 2013). There is no information about the influence of LPS exposure during the alveolarization period on morphological lung maturation and on immunological behavior in dependence of CD26 expression. Up to now, studies on the influence of inflammation on immune response in dependence of CD26 deficiency were carried out on adult rats (Kruschinski et al. 2005, 2008; Reinhold et al. 2009). Therefore, in this study, we tested the hypothesis that a 10-min LPS nebulization (1 µg LPS/2 ml NaCl), carried out for 7 days from dpp 3 to dpp 9, leads to a stronger pronounced delay in morphological lung development combined with an additional weak and different immune reaction in CD26-deficient lungs.

## Material and methods

### Animals

We used wild-type F344/Ztm rats (WT) and CD26/DPP4-deficient mutant rats (F344/Crl(Wiga)SvH-Dpp4m) without CD26/DPP4-activity and CD26/DPP4-expression (Karl et al. 2003). Rats and their litters were maintained in the Central Animal Facility of Hannover Medical School according to recommendations of the Federation of European Laboratory Animal Science Associations (FELASA) (Rehbinder et al. 1996). Animal care procedures and research had been authorized by the review board of the Landesamt für Verbraucherschutz und Lebensmittelsicherheit (06/1078, LAVES; Oldenburg, Germany) and were carried out according to international guidelines on the use of laboratory animals.

### Experimental schedule

Rat pups of litters were put in a closed glass chamber and inhaled low doses of LPS (1 µg LPS/2 ml NaCl) (Gerhold et al. 2003), which was distributed with a nebulizer (Pari LC Star (Pari, Starmberg, Germany) for 10 min on each day over a period of 7 days starting on day 3 postpartum (dpp3). Controls received no LPS.

### Processing of lungs

After random selection of healthy pups (controls) and pups exposed to LPS (*Escherichia coli*, serotype 0127:B8, Sigma, Darmstadt, Germany), the young animals of both substrains were killed on days postpartum (dpp) 7, 10, and 14 to investigate their lungs. Deep anesthesia was achieved by intraperitoneal application of a mixture of different concentrations and volumes of Medetomidin (DomitorVR 1 mg/mL, Pfizer, Karlsruhe, Germany) and Ketanest (KetaminVR 100 mg/mL, Dr. E. Graub AG, Bern, Switzerland) depending on the postnatal stage as already described (Wagener et al. 2020). Pups were killed by aortic exsanguination.

Lungs of the following postnatal age groups were investigated:

1. 7-day-old pups, which are in the alveolarization phase (Burri 2006)
2. 10-day-old pups, which are less sensitive to stress (stress non responsive period) (Sapolsky and Meaney 1986).
3. 14-day-old pups, which have finished the bulk alveolarization period (Tschanz et al. 2014)

### Histology — Stereology

After insertion and fastening of a cannula via the larynx into the trachea, the thorax was opened, and the heart–lung-blocks were taken out. Subsequently, the heart, thymus, and lymph nodes were removed.

Both lungs of each group were instilled with a mixture of cryo-gel “Tissue-Tec” (OCT, Torrance, CA, USA)/PBS (1:3) via gravitation (hydrostatic pressure 20 cm H_2_O) in a special instillation device. After tying off the trachea, the lung volume was determined using the fluid displacement method (Scherle 1970). Afterward, both lungs were frozen on dry ice.

Lung volume and body weight were determined and stated additionally as lung volume to body weight ratio as a measure, which was used for lung growth, particularly in preterm infants (De Paepe et al. 2014, 2005).

Using age-appropriate section protocols, the whole frozen lung blocks were completely cut from apical to basal to obtain 10 different cutting depth planes, which were all equally spaced from one another and comparable independent of age. Therefore, the 10-µm cuts per animal used for evaluation were at least 560 µm apart. Ten-micrometer-thick cryosections from the different cutting depths were selected randomly for evaluation. 

Sectioning of each lung block was carried out using a Cryostat (Leica CM 3050S, München, Germany). After staining with hematoxylin–eosin, scanning of test fields was carried out on a Nikon Eclipse 80i microscope (Tokyo, Japan) connected to a digital camera using the Stereo Investigator Version 6 software (Micro Bright Field, Vermont, USA) as described before (Hupa et al. 2014). According to the guidelines for standardized quantitative Assessment of Lung Structure (Hsia et al. 2010), the following parameters were determined using the point and intersection counting as described before (Hupa et al. 2014; Schmiedl et al. 2017; Wagener et al. 2020):

1. Volume densities of parenchyma (*V*_*V*_(par/par + nonpar)), counting test points, which hit alveoli, septa, small vessels, ductus alveolares (par) and arteries, venes, bronchi, bronchioli (nonpar) and using the formula: *P*(par)/*P*(par) + *P*(nonpar) × 100
2. Volume densities of septa (*V*_*V*_(septa/par)), counting test points, which hit alveolar septa (septa) and septa, ductus alveolares and alveoli (par) and using the formula: *P*(septa)/*P*(par) × 100
3. Volume densities of parenchymal airspaces (*V*_*V*_(airspace/par)) counting test points, which hit airspaces of alveoli, sacculi, and ductus alveolares (parenchymal airspaces) as well as septa, ductus alveolares and alveoli (par) and using the formula: *P*(airspace)/*P*(par) × 100
4. Surface densities (*S*_*V*_) of septa (*S*_*V*_ (septa/par)) counting test lines, which transect the surface of septa (intersections, *I*) and test lines which hit the parenchyma and using the formula: 2 × IS_septa_/P(par) × 1/2 length × ½ length of test line
5. The volume to surface ratio of septa V_S_-ratio_septa_ (mean septal wall thickness) counting the test points, which hit septa (alveolar epithelium, connective tissue, capillaries) and test lines, which transect the surface of septa (at least alveolar epithelium) and using the formula: *P*(septa) × ½ length of test line/2 × *I*
6. Mean linear intercepts (*MLI*) of airspaces as a measure for the mean free distances in airspaces by counting test points which hit the alveoli and ductus alveolares and intersections with the alveoli and ductus alveolares and using the formula: Lm = 4 × *V*(airpace)/*S*(alv, sac)

*V*_*V*_ and *S*_*V*_ are related to the parenchyma as reference space, which could also be subjected to alterations. Therefore, both parameters were applied to the total lung volume (Hupa et al. 2014; Schmiedl et al. 2017). To get information about total volume and surface of septa as well as about total volume of air spaces, we used the following formula:

$$V\left(\mathrm{septa};\mathrm{lung}\right)\left(\mathrm{cm}^3\right)=V_V(\mathrm{septa}/\mathrm{par})\times V_V(\mathrm{par}/\mathrm{par}+\mathrm{nonpar})\times V_{\mathrm{lung}}.$$

$$V\left(\mathrm{airspace};\mathrm{lung}\right)\left(\mathrm{cm}^3\right)=V_V(\mathrm{airspace}/\mathrm{par})\times V_V(\mathrm{par}/\mathrm{par}+\mathrm{nonpar})\times V_{\mathrm{lung}}.$$

$$S\left(\mathrm{septa};\mathrm{lung}\right)\left(\mathrm{cm}^2\right)=S_V(\mathrm{septa}/\mathrm{par})\times V_V(\mathrm{par}/\mathrm{par}+\mathrm{nonpar})\times V_{\mathrm{lung}}.$$

### Detection of inflammatory cells

Inflammatory cells such as neutrophils and macrophages express the β2-integrin CD11b as surface marker. Therefore, 10-µm sections also obtained from the different cutting depths were immunostained with the avidin biotin complex method. A monoclonal mouse anti-rat CD11b antibody (clone WT-5, PharMingen, San Diego, USA) was applied as primary antibody (1:100) and a biotin-conjugated affinity purified antibody (goat anti-rat IgG, Chemicon International Europe, Hampshire, UK; 1:500) as secondary antibody. Incubation in DAB (diaminobenzidine, Perhydrol, 30%, Merck, Darmstadt, Germany) substrate solution as chromogen led to a brown color of the detected inflammatory cells. The processing steps comprised blocking of endogenous peroxidase using 3% H_2_O_2_/phosphate-buffered saline (PBS) solution and incubation in avidin-D solution (Biotin/Avidin blocking kit SP-2001, Vector Laboratories, Burlinghame, USA). After rinsing in PBS-Tween solution and incubation in biotin solution (Biotin/Avidin blocking kit SP-2001, Vector Laboratories), sections were incubated with the primary antibody (1:00) overnight. Incubation with the secondary antibody (1:500) followed after additional rinsing for 30 min, followed by rinsing and incubation in peroxidase conjugated streptavidin solution (Jackson ImmunoResearch Europe Ltd., Newmarket, UK) for 15 min. After rinsing, the sections were incubated with DAB solution for 10 min and were rinsed several times (Wagener et al. 2020).

Immunostained inflammatory cells were counted in two sections per lung in 200–300 test fields (size of the test frame: 100 × 100 µm) with a constant interval of 400 µm between the test fields using the stereo investigator system (Micro-BrightField). Data were expressed as cell profile number per square millimeter (Wagener et al. 2020). However, this parameter was evaluated in a single section and has therefore some limitations. No statement is possible about the cell number, because the appearance of cell profiles in a single section was influenced by cellular alterations such as hypertrophy or shrinkage or swelling. However, this parameter read as cell profile density was only used to obtain some information about LPS-dependent influx of inflammatory cells.

The inflammatory status was determined on lung sections of 7- and 10-day-old pups (*n* = 3, each).

### FACS

The whole lung was homogenized using 50 ml phosphate-buffered saline (PBS; Seromed, Berlin, Germany) with 1% bovine serum albumin (BSA, Merck, Darmstadt, Germany) and 0.1% sodium acid (Sigma) as rinsing solution. After different centrifugation and resuspension steps, incubation with 20 ml erythrocytes lysis buffer (Schwinzer-lysis) followed. A suspension of 1 million cells per labeling was filled up with 100 µl of rinsing solution (PBS + 1%BSA + 0.1% sodium azide), and each transferred to a well of a microtiter plate (Greiner, Solingen Germany). FACS analyses were carried out using a FACSCanto flow cytometer (BD FACSCanto, Heidelberg, Germany), as already described (Skripuletz et al. 2007). Briefly, commercially available antibodies of different specificity (all from AbD Serotec, Duesseldorf, Germany) were used to analyze the different leucocyte and lymphocyte subpopulations, shown in Table 1. The dilutions of primary and secondary antibodies are listed in Table 2. The medium was transferred to FACS tubes filled with 300 µl measuring buffer. At least 50,000 events were counted in the mononuclear cell gate, defined previously, and the results were expressed as a proportion of lung leucocytes (Kruschinski et al. 2005; Skripuletz et al. 2007).

**Table 1:** Primary antibodies used for FACS-Analysis

Antibody	Antigen-specificity	Subpopulation
R73	T cell rezeptor of rat	T lymphocytes
Ox12	Immunoglobulin M of rat	B lymphocytes
Ox39	IL-2 receptor (CD25) of rat	Activated T lymphocytes
W3/W25	CD4 of rat	CD4^+^ T cells
Ox8	CD8 of rat	CD8^+^ T cells
ED9	CD172 of rat	monocytes /macrophages

**Table 2:** Primary antibodies used for FACS-analysis

Primary antibody/dilution	Secondary antibody/dilution
Ox 39/1:100	Phycoerythrin conjugated (kappa PE. Danova, Hamburg, Germany)/1:50
Ox12 FITC conjugated/1:100	–
Ox8 FITC conjugated/1:500	–
R73 biotinylated/1:50	Per-CP peridinin-chlorophyll-protein-complex (R&D Systems, USA)/1:500
W3/25 APC conjugated/1:200	–

The evaluation of the FACS data was carried out with the computer program Cell Quest (Becton Dickinson, San Jose, CA, USA). The ratios of the cells were displayed graphically in a dot plot graph. Depending on the cell size and granularity, the cell populations of lymphocytes, monocytes, and granulocytes were differentiated from each other, and cell cluster of mononuclear cells (lymphocytes and monocytes) was gated and selected from other cells. After selection of lymphocytes, their subpopulations were gated and differentiated based on the fluorescence intensity of the labeled cells. Single-color fluorescence was measured and evaluated in a fluorescence histogram. Two-color fluorescence was measured and evaluated in the dot plot graph. The basic fluorescence of cells when excited with light (autofluorescence) was differentiated from the antibody-mediated fluorescence and excluded. Only the antibody-mediated fluorescence was marked. Only these signals within the marked area (M1) were rated as positive and taken into account for evaluation (Pabst et al. 2003).

## Statistics

The entire graphics were created with the computer program “GraphPad Prism 6.07.” In the figures and tables, all data were plotted as arithmetic mean ± standard deviation (SD), if not indicated otherwise.

The calculations were done with the statistics program “Stat View 5.0” for windows (SAS Institute Inc., Cary, North Carolina, USA). Statistical differences were determined with the two-factorial ANOVA (two-way analysis of variance) with the factors strain and treatment as discriminatory factors. When statistically significant, starting at *p* < 0.05, a separation was made to the one-way analysis of variance (ANOVA) followed by a Fisher-PLSD post hoc analysis.

## Results

### Body weight and lung volume

#### Controls

Both substrains exhibited a comparable body weight on dpp 7, which continuously increased on dpp 10 and 14 independent of the substrain. Lung volume in both substrains also increased; however, it was significantly higher in the CD26^+^ group compared to CD26^−^ pups on dpp 7 and dpp 10 (Table 3). The lung volume body weight ratio as additional measure of immaturity (De Paepe et al. 2005, 2014) was somewhat lower in CD26^−^ controls than in CD26^+^ pups (Table 3).

**Table 3:** Postnatal body weight (BW) and lung volume (LV)(mean ± SD)

Genotyp	Dpp 7			Dpp 10			Dpp 14		
	BW	LV	LV/BW	BW	LV	LV/BW	BW	LV	LV/BW
CD26^+^ control (*n* = 3/group)	11.0 ± 2.0 g	0.81 ± 0.06 ml*	0.074	14.3 ± 4.7 g	1.24 ± 0.05 ml*	0.087	20.3 ± 1.5 g	1.41 ± 0.010 ml*	0.070
CD26^+^ LPS exposed (*n* = 3/group)	12.5 ± 1.1 g	0.78 ± 0.03 ml*	0.062	14.0 ± 2.0 g	1.23 ± 0.05 ml*	0.088	19.9 ± 5.3 g	1.42 ± 0.06 ml	0.071
CD26^−^ control (*n* = 5/group)	10.1 ± 0.5 g	0.62 ± 0.08 ml	0.060	14.3 ± 0.9 g	0.85 ± 0.056 ml	0.059	21.0 ± 1.6 g	1.21 ± 0.05 ml*	0.058
CD26^−^ LPS exposed (*n* = 3/group)	10.5 ± 0.7 g	0.58 ± 0.03 ml	0.055	13.7 ± 0.7 g	0.80 ± 0.045 ml	0.058	22.0 ± 1.6 g	1.22 ± 0.035 ml	0.055

^*^p < 0.05 compared to CD26/DPP4^−^ pups

#### LPS exposure for 7 days

Chronically applied LPS resulted in a more pronounced significant increase of body weight in CD26^+^ pups than in CD26^−^ pups compared to controls only on dpp 7 (Table 3). Lung volume was not influenced by LPS on dpp 7, 10, and 14. The lung body weight ratio was only notably lower noteworthy on dpp 7 compared to controls independent of the substrain (Table 3).

ANOVA two-way analyses exhibited that body weight and lung volume were influenced by the treatment, but not by the genotype. However, the genotype had the same effect as the treatment-dependent values on dpp 7 (Table 4). The genotype affected the values on dpp 14 (Table 4).

**Table 4:** ANOVA two-way analyses, tabular results

	Body weight	Lung volume	Ssepta	Vsepta	Vairspace	MLI	Septal thickness
Dpp 7							
Genotype	n.s	n.s	0.0091	0.0012	0.0131	n.s	n.s
Treatment	0.0001	0.0001	0.0003	0.0168	n.s	0.0002	n.s
Interaction	0.0002	0.0002	n.s	n.s	n.s	n.s	n.s
Dpp 10							
Genotype	n.s	0.0001	0.0001	0.001	0.0001	0.1121	0.1324
Treatment	n.s	n.s	0.0139	0.0013	0.2173	0.0247	0.0088
Interaction	n.s	n.s	n.s	n.s	0.1183	0.1755	n.s
Dpp 14							
Genotype	0.0006	0.0001	0.0005	0.01	n.s	0.1962	n.s
Treatment	0.0318	n.s	n.s	00.0463	n.s	0.1572	n.s
Interaction	n.s	n.s	n.s	n.s	n.s	0.0663	n.s

### CD11 positive inflammatory cells in lung parenchyma

To obtain some information about the parenchymal inflammation, we carried out labeling with the surface marker ß_2-integrin CD11b.

#### Controls

**Fig. 1:**
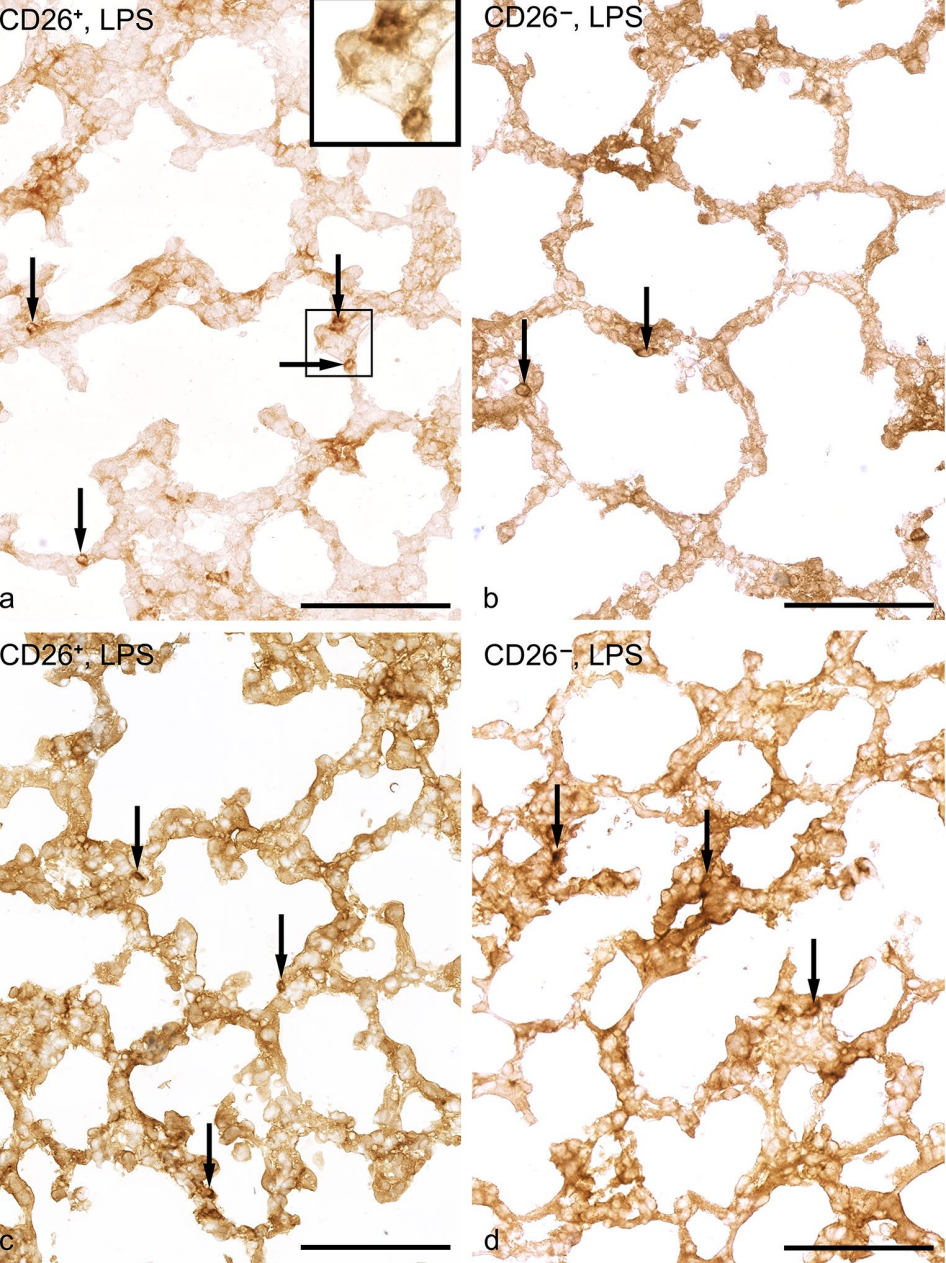
Immunohistochemically labeled inflammatory cells indicated by arrows using the avidin biotin complex method to detect the β2-integrin CD11b as surface marker. Inflammatory cells in lungs of 7- and 10-day-old lungs in both genotypes after LPS exposure are rarely found: **a** CD26^+^ dpp 7 (bar = 100 µm), **b** CD^−^ dpp 10 (bar = 100 µm), **c** CD26^+^, dpp7 (bar = 100 µm, **d** CD26^−^ dpp 10 (bar = 100 µm)

CD11 positive inflammatory cells were barely visible (Fig. 1). Using *cell profile density* as a quantitative parameter, only a few CD11-positive granulocytes and macrophages were determined. There were no subtype specific differences (Table 5).

**Table 5:** Cell profile densities of CD11b labeled inflammatory cells

Genotype		CD26/DPP4^+^		CD26/DPP4^−^	
mean ± SEM		cells/mm^2^		cells/mm^2^	
dpp	Inflammatory cells	Controls	LPS	Controls	LPS
7	Neutrophils	2.3 ± 0.4	14.7 ± 1.5*	2.6 ± 0.7	10.3 ± 3.7*
10	Macrophages	0.7 ± 0.5	2.0 ± 1.4	0.1 ± 0.0	0.5 ± 0.0
	Neutrophils	1.9 ± 0.1	16.5 ± 4.0*	3.1 ± 2.2	11.7 ± 0.2*
	Macrophages	0.4 ± 0.2	4.6 ± 0.7	0.5 ± 0.0	1.5 ± 1.5

^*^p < 0.05 compared to controls

*n* = 3/group

#### LPS exposure for 7 days

In the lungs of the animals exposed to LPS, there was a very mild inflammatory reaction with evidence of immigrated CD11b-positive macrophages and neutrophil granulocytes. However, in both subtypes, the cell profile density of macrophages increased only slightly in both groups (Fig. 1). Significantly increased cell profile densities of neutrophils were found in both groups on dpp 7 and dpp 10 without significant subtype differences (Table 5).

### Qualitative morphology

#### Controls

**Fig. 2:**
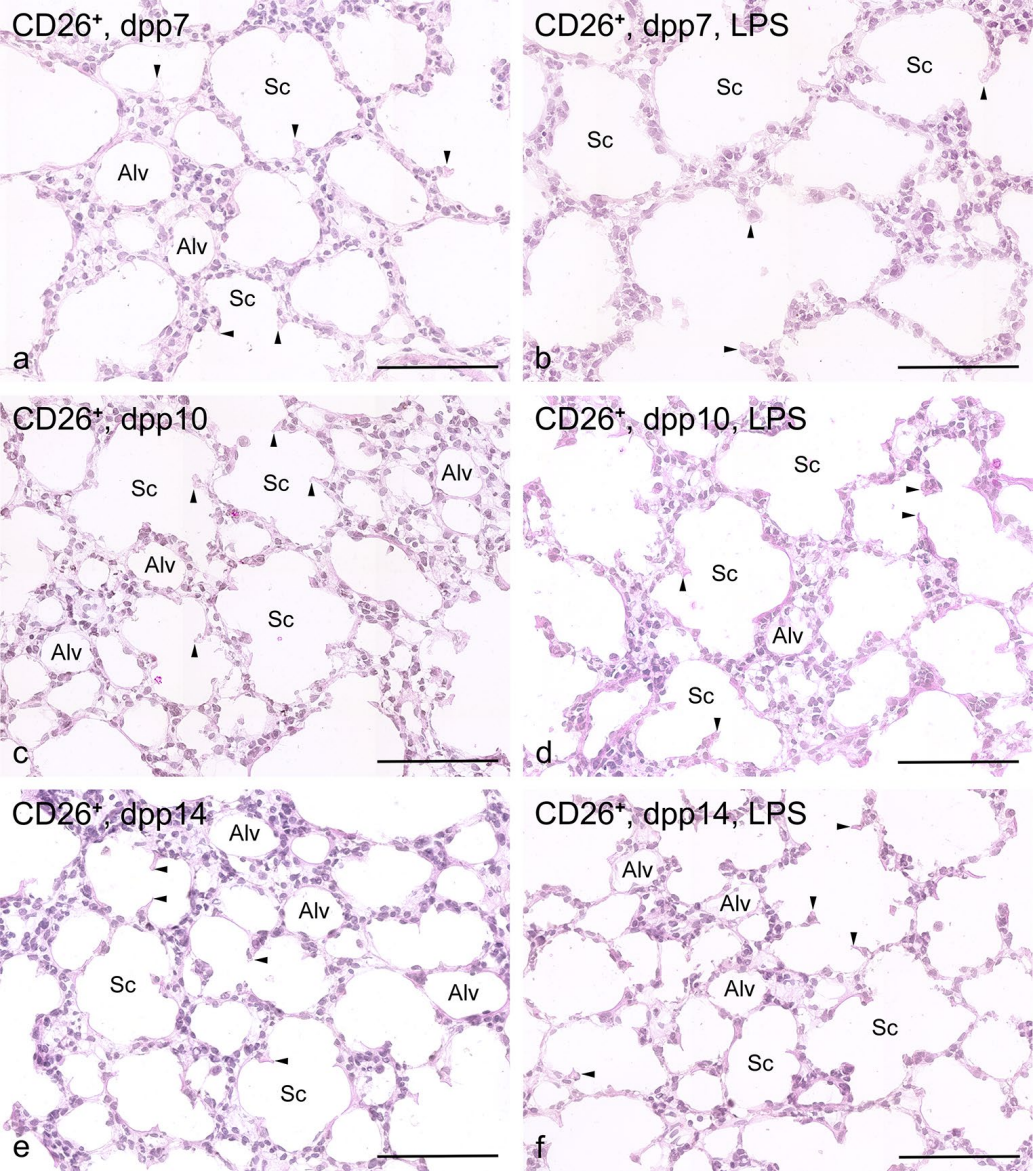
Morphological lung development without and with LPS exposure in CD26^+^ pups (bar = 100 µm). **a** Control, 7 dpp: lung parenchyma exhibit smooth-walled primary septa and numerous so called secondary septa ( arrow heads) are formed, extending into the expanding air spaces. (Alv = alveolus, Sc = Sacculus) **b** LPS exposure, 7 dpp: airspaces are still relatively large, and the sprouts of the secondary septa (arrow heads) are seen less frequently in lung parenchyma. Alveolar septa are structurally intact. (Alv = Alveolus, Sc = Sacculus). **c** Control, 10 dpp: multiple smaller airspaces formed by secondary septation (arrow heads). (Alv = Alveolus, Sc = Sacculus). **d** LPS exposure, 10 dpp: newly formed septa are visible, the number of sprouts (arrow heads) is less frequent, and no signs of destruction of the septa are recognizable. (Alv = Alveolus, Sc = Sacculus). **e** Control, 14 dpp: numerous small alveoli (Alv) in the lung parenchyma are visible. (Alv = Alveolus, Sc = Sacculus, arrow head = secondary septa). **f** LPS exposure, 14 dpp: a further increase in newly formed intact alveoli (Alv) is seen. (Alv = Alveolus, Sc = Sacculus, arrow head = secondary septa)

**Fig. 3:**
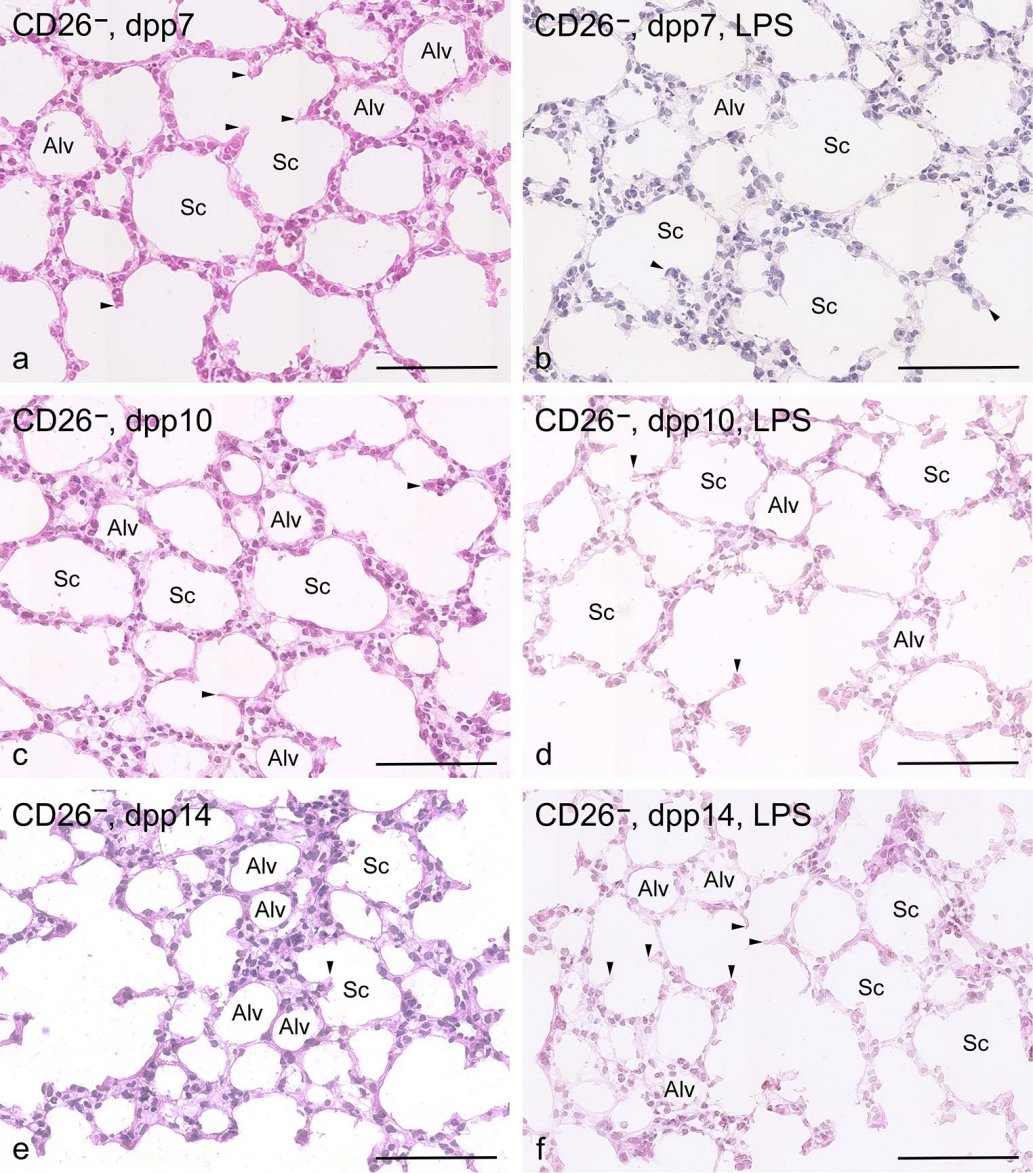
Morphological lung development without and with LPS exposure in CD26^−^ lungs (bar = 100µm): **a** Control, 7 dpp: sacculi and some so-called secondary septa are visible. (Alv = Alveolus, Sc = Sacculus, arrow head = secondary septa). **b** LPS exposure, 7 dpp: sacculi predominate in the lung parenchyma. Alveoli are sporadically seen. Alveolar septa are structurally intact. (Alv = Alveolus, Sc = Sacculus, arrow head = secondary septa), **c** Control, 10 dpp: newly formed alveoli are found between sacculi. No signs of destruction of the septa are recognizable. (Alv = Alveolus, Sc = Sacculus, arrow head = secondary septa). **d** LPS exposure, 10 dpp: newly formed alveoli are sporadically recognizable. **e** Control, 14 dpp: many alveoli are seen. (Alv = Alveolus, Sc = Sacculus, arrow head = secondary septa). **f** LPS exposure, 14dpp: several larger alveoli and sacculi with sprouting short secondary septa are visible. (Alv = Alveolus, Sc = Sacculus, arrow head = secondary septa)

Wild-type controls exhibited a morphology corresponding to the stage of development. Qualitatively, only slight differences between CD26^+^ and CD26^−^ pups were visible. On dpp 7, previously smooth-walled primary septa were sprouting in many places (Figs. 2a and 3a). From these “buds,” the so-called secondary septa were visible, drawn into the expanding air spaces and thus represented the boundary of the future alveoli. On dpp 10, the rat lungs were in the middle of alveolarization (Figs. 2c and 3c). Compared to dpp 7, the bronchial tree had branched out more, so that many new air spaces had arisen. On dpp 14, at the end of the bulk alveolarization phase, a lot of small alveoli were visible in the lungs. The lung parenchyma contained numerous alveoli (Figs. 2e and 3e).

#### LPS exposure for 7 days

On dpp 7, the airspaces were still relatively large, and the sprouts of the secondary septa were seen less frequently compared to controls independent of the CD26 expression (Figs. 2a,b and 3a,b). However, the septa looked intact. Newly formed septa had not been destroyed. Qualitative differences in the degree of maturation were barely visible between the two substrains. The terminal airspaces showed less sprouts as a sign of the beginning of septation and seemed to be somewhat larger in the CD26-deficient group (Figs. 2b and 3b). On dpp 10, newly formed alveoli were seen in both strains, and the number of sprouts had increased (Figs. 2d and 3d). Also, no signs of destruction of the septa were visible. Lungs on dpp 14 showed a further increase in newly formed alveoli without recognizable substrain-specific differences (Figs. 2f and 3f).

### Bronchi and peribronchial spaces

Controls of both groups showed no inflammatory cells around bronchi or vessels.

After LPS exposure, clearly visible signs of inflammation in the bronchial wall or in the peribronchial space were not detected in both groups on dpp 7or on dpp 10.

### Stereological parameters

#### Controls

**Fig. 4:**
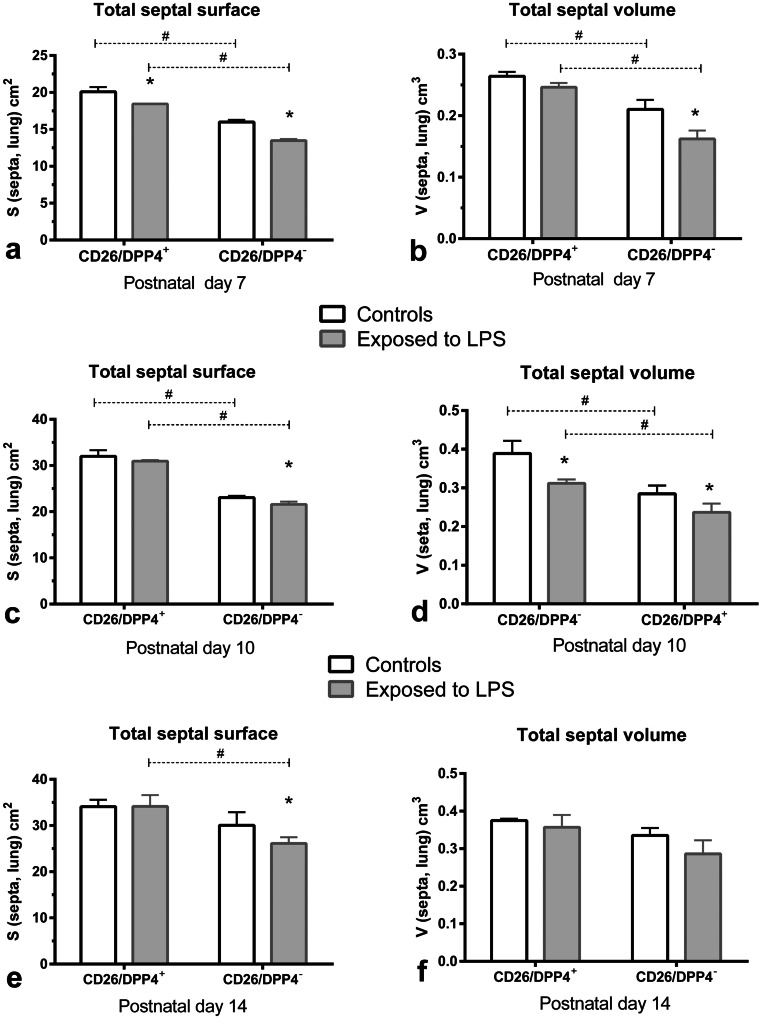
Stereologically determined parameters exhibiting morphological lung maturation, given as mean ± SD: total septal surface, serving as a parameter indicating alveolar septation as an indirect measure for alveolarization: **a** pups of dpp7 (*n* = 3/group), **c** pups of dpp10 (*n* = 3/group), **e** pups of dpp 14 (*n* = 3/group); total septal volume, taken as a parameter for the volume of primary and newly formed septa, without considering their thickness: **b** pups of dpp7 (*n* = 3/group), **d** pups of dpp10 (*n* = 3/group), **f** pups of dpp 14 (*n* = 3/group)

**Fig. 5:**
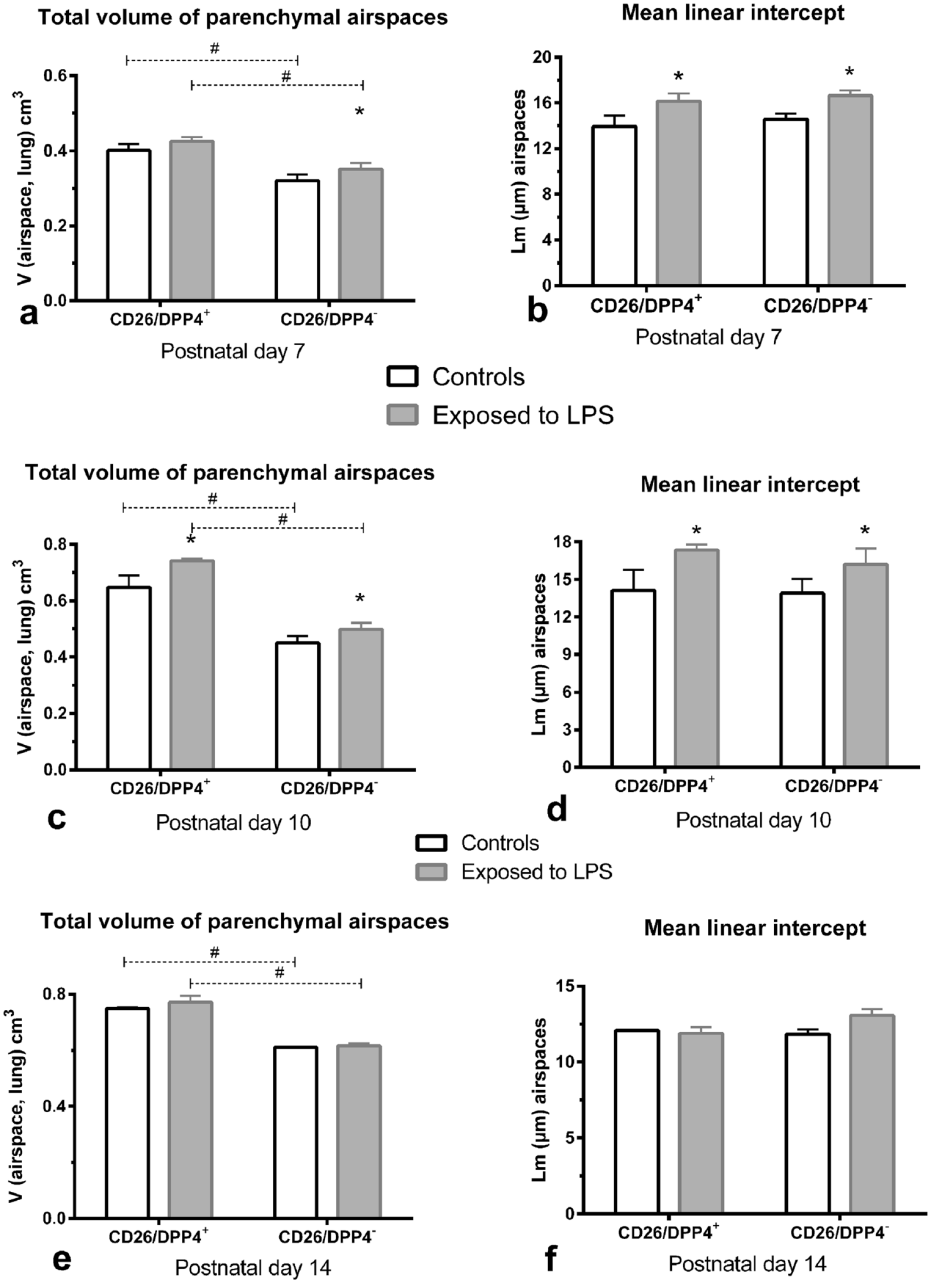
Total volume of airspaces of sacculi/alveoli, serving as a parameter for enlargement or reduction of airspaces, given as mean ± SD: **a** pups of dpp7 (*n* = 3/group), **c** pups of dpp10 (*n* = 3/group), **e** pups of dpp 14 (*n* = 3/group). Size of saccui/alveoli, a size parameter independent of the reference space **b** pups of dpp 7 (*n* = 3/group), **d** pups of dpp 10 (*n* = 3/group), **f** pups of dpp 14 (*n* = 3/group)

The results showing significantly lower values of *S*(septa, lung), *V*(septa, lung), *V*(airspace, lung) in lungs of CD26^−^ pups compared to CD26^+^ pups on dpp 7 and 10 should be mentioned here (Figs. 4 and 5). These have already been published (Wagener et al. 2020).

#### LPS exposure for 7 days

The *S*(septa, lung) as an indirect parameter for alveolarization was significantly lower on dpp 7 and in the CD26^−^ group also on dpp 10 in both substrains compared to controls, meaning that LPS reduced the formation of secondary septa. On dpp 14, comparable *S*(septa, lung) were determined in CD26^+^ pups; however, in CD26^−^ pups, the reduced total septal surface still remained significantly lower (Fig. 4a, c, e).

The *V*(septa, lung) was significantly lower compared to control values on dpp 10 in the CD26^+^ group and on dpp 7 and 10 in the CD26^−^ group. Both substrains showed similar values compared to controls on dpp 14 (Fig. 4b, d, f).

The volume of parenchymal airspaces was higher compared to controls, but reached significance only in the CD26^−^ group (Fig. 5a, c, e). Significantly lower values were determined on dpp 10 in both groups compared to controls. No differences were seen on dpp 14. MLI, a measure of the mean free distances in airspaces, was higher after LPS exposure in both substrains on dpp 7 and dpp 10 (Fig. 5b, d, f).

The arithmetic mean septal thickness was not influenced by LPS exposure compared to controls. The arithmetic mean septal thickness was 13.3 ± 0.4 µm in the CD26^+^ group compared to 13.2 ± 0.8 µm on dpp 7, 10.7 ± 0.4 µm compared to 12.2 ± 0.6 µm on dpp 10, and 10.5 ± 0.5 μm compared to 11.0 ± 0.1 µm on dpp 14. In the CD26-deficient group, the arithmetic mean septal thickness amounted to 12.5 ± 1.0 µm compared to 13.1 ± 1.1 µm on dpp 7, to 11.4 ± 1.1 µm compared to 12.4 ± 0.9 µm on dpp 10 and to 11.0 ± 0.6 µm compared to 11.1 ± 0.1 µm on dpp 14.

Looking at the tabular results of the two-way ANOVA tests (Table 4), we could summarize that the parameters Ssepta and Vsepta were significantly influenced by genotype and treatment on dpp 7 and dpp 10. On dpp 14, only the genotype affected the results significantly (Table 4). However, the impact of the treatment was independent of the genotype (Table 4). Vairspace was significantly influenced by the genotype on dpp 7 and dpp 14, and MLI was significantly influenced by the treatment on dpp 7 and dpp 10, the arithmetic mean septal thickness by the treatment on dpp 10.

Summarizing our results, LPS-induced effects were detectable in both strains on the dpp 7. This showed up in the form of a reduced septa surface and septal volume fraction, as well as an increase in the absolute volume of the air space and an increase of MLI as a measure of the mean free distances in airspaces. The substrain-specific differences already described for the control animals remained. Also on dpp 10, LPS effects were demonstrated in both strains on postnatal morphological pulmonary maturation as a decrease in the absolute septal volume, a related increase in the absolute volume of the airspace, increased MLI as a measure of the mean free distances in airspaces and narrower septa. The LPS-dependent differences remained in terms of volumes and septa surface. However, LPS-induced changes were no longer detectable in the wild-type animals on the dpp 14, although a reduced septal surface was still detectable in the CD26-deficient animals.

### Characterization of immune cells

#### Controls

To obtain some information about the influence of CD26 on the immunological status in both substrains, we determined the composition of immunological cells using FACS analyses and stated the results as percentage of total leucocytes in lung homogenates. Absolute cell numbers are listed in Table 6. Because of the variation in absolute cell numbers, the percentage of immune cells was used for comparisons (Table 7).

**Table 6:** Absolute immune cell numbers in the lung during alveolarization

**Absolute numbers of immune cells**	CD26/DPP4^+^						CD26/DPP4^−^					
	dpp	7	10		14		dpp	7	10		14	
**X ± SD** *n* = 5/group	Control	LPS	Control	LPS	Control	LPS	Control	LPS	Control	LPS	Control	LPS
Total cell count	3.00 ± 0.4 × 10^6^	4.25 ± 0.3 × 10^6^	6.88 ± 0.4 × 10^6^	3.42 ± 0.6 × 10^6^	9.69 ± 0.6 × 10^6^	3.05 ± 0.2 × 10^6^	2.54 ± 0.7 × 10^6^	2.83 ± 0.3 × 10^6^	4.93 ± 0.6 × 10^6^	4.09 ± 0.6 × 10^6^	8.84 ± 1.4 × 10^6^	2.39 ± 0.2 × 10^6^
Monocytes	0.52 ± 0.08 × 10^6^	0.63 ± 0.04 × 10^6^	1.23 ± 0.06 × 10^6^	0.58 ± 0.12 × 10^6^	1.56 ± 0.38 × 10^6^	0.54 ± 0.05 × 10^6^	0.27 ± 0.05 × 10^6^	0.40 ± 0.07 × 10^6^	0.61 ± 0.05 × 10^6^	0.70 ± 0.13 × 10^6^	1.01 ± 0.2 × 10^6^	0.39 ± 0.05 × 10^6^
Lymphocytes	1.48 ± 0.04 × 10^6^	2.0 ± 0.2 × 10^6^	3.72 ± 0.3 × 10^6^	1.85 ± 0.4 × 10^6^	5.26 ± 0.7 × 10^6^	1.86 ± 0.1 × 10^6^	1.31 ± 0.5 × 10^6^	1.31 ± 0.2 × 10^6^	2.15 ± 0.2 × 10^6^	2.34 ± 0.3 × 10^6^	4.29 ± 0.8 × 10^6^	1.49 ± 0.1 × 10^6^
B lymphcytes	0.14 ± 0.03 × 10^6^	0.37 ± 0.02 × 10^6^	0.40 ± 0.05 × 10^6^	0.30 ± 0.14 × 10^6^	0.52 ± 0.13 × 10^6^	0.43 ± 0.11 × 10^6^	0.10 ± 0.04 × 10^6^	0.22. ± 0.05 × 10^6^	0.22 ± 0.05 × 10^6^	0.34 ± 0.07 × 10^6^	0.58 ± 0.09 × 10^6^	0.35 ± 0.07 × 10^6^
T lymphocytes	0.45 ± 0.04 × 10^6^	0.70 ± 0.09 × 10^6^	1.29 ± 0.19 × 10^6^	0.63 ± 0.14 × 10^6^	1.97 ± 0.28 × 10^6^	0.67 ± 0.09 × 10^6^	0.52 ± 0.16 × 10^6^	0.53. ± 0.09 × 10^6^	0.79 ± 0.19 × 10^6^	0.94 ± 0.05 × 10^6^	2.07 ± 0.45 × 10^6^	0.58 ± 0.07 × 10^6^
CD8 + cells	0.14 ± 0.01 × 10^6^	0.20 ± 0.02 × 10^6^	0.33 ± 0.04 × 10^6^	0.17 ± 0.05 × 10^6^	0.5 ± 0.09 × 10^6^	0.18 ± 0.02 × 10^6^	0.14 ± 0.03 × 10^6^	0.13 ± 0.03 × 10^6^	0.26 ± 0.05 × 10^6^	0.23 ± 0.02 × 10^6^	0.46 ± 0.11 × 10^6^	0.08 ± 0.01 × 10^6^
CD4 + cells	0.22 ± 0.02 × 10^6^	0.39 ± 0.04 × 10^6^	0.72 ± 0.12 × 10^6^	0.4 ± 0.08 × 10^6^	1.06 ± 0.18 × 10^6^	0.44 ± 0.05 × 10^6^	0.27 ± 0.08 × 10^6^	0.31 ± 0.05 × 10^6^	0.45 ± 0.18 × 10^6^	0.64 ± 0.03 × 10^6^	1.38 ± 0.31 × 10^6^	0.19 ± 0.03 × 10^6^
CD4^ +^ CD25 ^+^T cells	2.07 ± 0.99 × 10^4^	1.71 ± 0.22 × 10^4^	3.14 ± 0.64 × 10^4^	1.13 ± 0.31 × 10^4^	5.52 ± 3.48 × 10^4^	0.90 ± 0.19 × 10^4^	0.27 ± 0.05 × 10^4^	01.48 ± 0.37 × 10^4^	0.61 ± 0.05 × 10^4^	1.88 ± 0.13 × 10^4^	1.01 ± 0.2 × 10^4^	0.96 ± 0.08 × 10^4^

**Table 7:** Percentage of immunological cells in the lung during alveolarization

Leucocytes	CD26/DPP4^+^						CD26/DPP4^-^					
	dpp	7	10		14		dpp	7	10		14	
n = 5/group	Control	LPS	Control	LPS	Control	LPS	Control	LPS	Control	LPS	Control	LPS
Monocytes % of leucocytes	17 ± 2	15 ± 3	18 ± 1	17 ± 2	16 ± 2	18 ± 2	11 ± 2#	14 ± 2*	12 ± 2#	17 ± 3*	11 ± 2#	16 ± 2*
Lymphocytes % of leucocytes	48 ± 2	48 ± 1	54 ± 2	54 ± 7	51 ± 2	61 ± 2*	49 ± 6	47 ± 5	44 ± 3#	57 ± 2*	48 ± 1	63 ± 2
B lymphocytes % of lymphocytes	10 ± 2	17 ± 2*	11 ± 1	16 ± 1*	10 ± 3	23 ± 1*	8 ± 1	10 ± 2	16 ± 3	10 ± 1#	14 ± 1#	23 ± 1*
T lymphocytes % of lymphocytes	30 ± 1	33 ± 5	35 ± 3	35 ± 2	38 ± 2	36 ± 2	40 ± 3#	41 ± 1#	37 ± 6	40 ± 3#	48 ± 3#	39 ± 1#
CD8^+^ T cells % of lymphocytes	9 ± 1	10 ± 1	9 ± 1	9 ± 1	11 ± 1	9 ± 1	10 ± 2	10 ± 0.5	12 ± 0.5	10 ± 0.5	11 ± 1	5 ± 2
CD4^+^ T cells % of lymphocytes	15 ± 2	20 ± 2*	19 ± 2	22 ± 1	22 ± 2	23 ± 1	21 ± 3#	24 ± 1	17 ± 2	27 ± 2*	32 ± 3#	25 ± 1*
CD4^+^CD25^+^ T cells % of lymphocytes	1.39 ± 0.55	0.85 ± 0.05*	0.83 ± 0.17	0.62 ± 0.07*	1.03 ± 0.07	0.48 ± 0.03*	1.31 ± 0.05#	1.12 ± 0.13*#	1.13 ± 0.07#	0.8 1 ± 0.12*#	1.00 ± 0.05#	0.64 ± 0.078*#

^#^Significance between strains

*Significance between control and LPS

The percentage of monocytes was significantly lower in controls of CD26 deficient rats.

Looking at the percentage of lymphocytes, no substrain or developmental dependent differences existed on dpp 7. However, lower percentages of lymphocytes were found on dpp 10 and dpp 14 in CD26^−^ pups reaching significance only on dpp 10 (Table 7). The B lymphocytes showed no strain-dependent differences. To check whether there is an increase in the portion of T cell subpopulations that may produce maturation-relevant cytokines, different T-cell subpopulations were studied. Interestingly, in the controls, we found remarkable higher values of T lymphocytes in CD26^−^ rats compared to CD26^+^ rats, reaching significance on dpp 7. Both groups showed comparable values of CD8^+^ T cells (Table 7). The percentage of CD4^+^ T cells was significantly higher in the CD26^−^ group on dpp 7 and dpp 14 (Table 7). The CD4^+^/CD25^+^ T cells containing the activated and the regulatory T cells were significantly higher on each postnatal day in the CD26^−^ compared to the CD26^+^ pups (Table 7). The percentage of monocytes was significantly lower in the CD26-deficient group on dpp 7, dpp 10, and dpp 14 compared to CD26^+^ pups. Thus, we found some strain-dependent differences in the population of leucocytes in controls.

#### LPS exposure for 7 days

In order to check whether the differential influence of LPS on lung morphogenesis is also seen in the leukocyte composition and thus the inflammatory reaction to the LPS stimulus, FACS analyses of lung homogenates were additionally performed.

The percentage of monocytes was influenced by LPS only on dpp 7 (Table 7).

When looking at all lymphocytes, no effects were found on the dpp 7. While on dpp 10 and dpp 14, significant increases in the percentage of lymphocytes were determined in both substrains. On dpp 10, there was an increase of 3% in wild-type rats and 13% in CD26-deficient rats. On dpp 14, the proportion of lymphocytes in wild-type rats increased by 11%, in CD26-deficient rats by 15% (Table 7).

We found a significant increase of B lymphocytes on dpp7 and dpp 14 in both substrains and on dpp 10 and dpp 14 in CD26^+^ pups. LPS did not influence T lymphocytes in their percentages. However, compared to CD26^+^ pups, the percentage of T lymphocytes was significantly higher in the CD26^−^ pups on each dpp (Table 7). Compared to controls, the percentage of CD4^+^ T-cells was significantly increased on dpp 7 in the CD26^+^ group and on dpp 10 in the CD26^−^ group. No effects were detectable on dpp 14 (Table 7). The CD4^+^/CD25^+^ T-cells, which contained regulatory T-cells, were significantly lower compared to controls in the CD26^+^ group not only on dpp 7, but also on dpp 10 and dpp 14 independent of the substrain.

ANOVA two-way analyses showed that the percentage of monocytes and T-cells was influenced by the genotype during all postnatal developmental stages. Treatment influenced the percentage of CD8^+^ and CD4^+^ cells at all time points investigated (Table 8). Combined effects of genotype and treatment were seen for monocytes on dpp7 and dpp 10, for lymphocytes on dpp10, for B-cells on dpp7, for T-cells on dpp 14 (Table 8).

**Table 8:** ANOVA two-way analyses, tabular results

	Monocytes	Lymphocytes	B-cells	T-cells	CD8^+^ cells	CD4^+^ cells	CD4^+^CD25^+^ cells
Dpp 7							
Genotype	0.0001	n.s	0.024	0.0001	n.s	0.0004	0.205
Treatment	n.s	0.1328	0.009	n.s	0.0238	0.0043	0.025
Interaction	0.0001	n.s	n.s	n.s	0.2004	n.s	n.s
Dpp 10							
Genotype	0.0001	0.0761	0.0001	0.023	0.0928	0.03	0.0001
Treatment	0.002	0.0017	n.s	0.081	0.0516	0.0001	0.0001
Interaction	0.0001	0.0014	0.0001	n.s	0.0928	0.028	0.0001
Dpp 14							
Genotype	0.0057	n.s	0.152	0.0001	n.s	n.s	n.s
Treatment	0.0032	0.0002	0.0001	0.0001	0.03	0.0001	0.0008
Interaction	0.1309	0.1083	0.0141	0.0036	n.s	0.0001	n.s

### Validation of CD26 deficiency

The absence of CD26 in the CD26 deficient rat group has been already proved in several publications (Zientara et al. 2019; Karl et al. 2003; Kruschinski et al. 2005; Schade et al. 2008). To verify additionally the CD26 deficiency during postnatal development in lungs, we determined the content of CD26^+^ CD4^+^ cells (Table 9). In the deficient rat lungs, no CD26^+^ cells were found. LPS application did not change the CD26^+^ T cells in the CD26^+^ pups. In the CD26^−^ pups, the CD26-T cells were significantly increased compared to controls on dpp 10 and dpp 14 (Table 9).

**Table 9:** Percentage of CD 26^+^ T cells in the lung of CD26^+^ and CD26^−^ rats

% of lymphocytes	CD26/DPP4^+^						CD26/DPP4^−^					
	dpp	7	10			14	dpp	7	10			14
*n* = 5/group	Control	LPS	Control	LPS	Control	LPS	Control	LPS	Control	LPS	Control	LPS
CD26^+^ T cells	26.8 ± 2.1#	31.7 ± 2.1#*	29.9 ± 2.5*	32.2 ± 1.2#	33.1 ± 1.8#	33.5 ± 1.3#	0.0 ± 0.0#	0.0 ± 0.0#	0.0 ± 0.0#	0.0 ± 0.0#	0.0 ± 0.0#	0.0 ± 0.0#
CD26^−^ T cells	1.4 ± 0.4	2.5 ± 0.5	1.2 ± 0.1	1.0 ± 0.3	2.1 ± 0.4	1.2 ± 0.2	37.7 ± 3.1	38.3 ± 1.0	32.3 ± 1.2	40.8 ± 1.5*	33.5 ± 1.3	38.0 ± 1.3*

^#^Significance between strains

*Significance between control and LPS

## Discussion

Compared to CD26/DPP4^+^ wild types, CD26 deficiency led to comparable body weights, but to significantly lower lung volumes and significantly lower total septal surface, septal volume, and parenchymal airspace volume on dpp 7 and dpp 10. However, the lung (weight) volume to body weight ratio, determining the lung growth in relation to body weight as an indirect measure for immature lungs (De Paepe et al. 2014, 2005), exhibited no significant LPS-dependent differences during postnatal development. Thus, our LPS model does not lead to remarkable delay in lung growth.

Regarding the immune status in CD26-deficient pups, significantly higher percentages of T cells and CD4^+^ T cells were found on dpp 7 as well as significantly higher percentages of CD4^+^CD25^+^ T cells and significantly lower percentages of monocytes.

The maturing lung is particularly at risk of being damaged by external influences. In addition to the structural immaturity of the respiratory system, the immune system is not yet fully developed in the fetal compared to the adult lung (Martin and Frevert 2005; Anderson 2000). The nebulization of LPS dissolved in physiological common salt as application method enables precise dosing. It also represents a very gentle method for the animals, since they hardly need to be manipulated, and they can remain together as a litter. This method is therefore well suited for multiple applications over a period of several days. The disadvantage of this form of application is that the aerosol may not only be absorbed via the lungs, but over the entire body surface.

### Measurement of retarded lung maturation

To determine the lung volume, we used the water displacement method (Scherle 1970). Because of the small size of lungs, a lack of precision using the Archimedes principle was postulated (Pozarska et al. 2017). We always measured both lungs and accurately dried the lobes and dapped off the water droplets in the interlobar regions. Doing so, the variation of lung volume within the investigated groups was in a tolerable range, and the population coefficient of variation was in all cases lower than 10%.

There are different ways to analyze alveolarization. Often, the radial alveolar counts (RACs) were determined (Dasgupta et al. 2009; Dauger et al. 2003; Husain et al. 1998; Shrestha et al. 2019; Cooney and Thurlbeck 1982a). The RAC reflects the number of alveoli per unit area as a measure of complexity of the terminal respiratory unit (acinus). It gives no information about the total number of alveoli or alveoli per unit volume (Cooney and Thurlbeck 1982b, 1982a). The RAC method shows an indirect correlation to numbers of alveoli (Herring et al. 2014). Mean linear intercepts (MLI) were also used to determine lung maturation, because this method may be suitable for determining the size of airspaces (Husain et al. 1998; Manzano et al. 2014; Tiono et al. 2019). However, MLI do not really measure alveolar size as often argued (Ochs 2014). MLI measure the mean free distance between walls of acinar spaces (Knudsen et al. 2010). Furthermore, values of MLI depended on the degree of normal lung inflation (Ochs 2014). To determine morphological lung maturation, we therefore used single-section parameters which characterize predominantly the parenchymal compartments. Using the point and intersection counting method together with the known lung volume, the determined parameters give sufficient information about the total amount of airspace and septa as well as the total septal surface area (Ochs 2014; Schmiedl et al. 2017; Wagener et al. 2020; Hupa et al. 2014). The increase of septal surface is an indirect indication for new secondary septa and therefore of alveolarization. Furthermore, the surface area correlates very well with the total gestation age (Cooney and Thurlbeck 1982b). The determination of the total septal volume and the total airspace volume in combination with the total surface area permits estimation of the architecture of the developing lung parenchyma.

However, our investigations were carried out on frozen lungs and frozen sections. Therefore, we cannot rule out the influence of strain and/or treatment-dependent shrinkage on the parameters used.

### Determining the immune status

We presented the percentage distribution of immune cells to avoid inaccuracies caused by problems in the determination of total numbers of cells resulting in great variations. In the present work, FACS analyses were firstly carried out on the lungs of rat juveniles to also compare substrain-dependent differences.

### CD26 and immune status

CD26 cleaves not only several cytokines and chemokines as well as peptide hormones involved in the regulation of the immune system but also modulates lymphocyte function (Broxmeyer et al. 2016;Proost et al. 2000;Klemann et al. 2016;Van et al. 1999;Shao et al. 2020). So, it is involved in the costimulation of T cells (Ohnuma et al. 2008) and may influence transepithelial T cell migration (Shao et al. 2020). The degree of CD26 expression on CD4^+^ T cells additionally influences the behavior of these cells (Shao et al. 2020). A higher expression of CD26 results in a Th1-like helper phenotype combined with the secretion of inflammatory cytokines such as interferon γ (INFγ) and interleukin 2 (IL2) and activation of macrophages, CD8 cells, whereas low expression is accompanied with the TH2-phenotype combined with a secretion of IL 4, IL 5, IL 10, TGF β, and activation of CD4^+^ cells with stimulation of B lymphocytes (Reinhold et al. 1997; Shao et al. 2020). Looking at the immune cells, these findings are partly comparable with our results. So, we evaluated generally partly significantly higher percentages of CD4^+^ T cells in the CD26^−^ group during postnatal development.

Furthermore, CD26 was also seen on the surface of other immune cells such as B cells, dendritic cells, and macrophages as summarized by Shao et al. (Shao et al. 2020; Buhling et al. 1995). Preliminary investigations by our group had shown that there are differences in the composition of immune cells in adult animals in a strain comparison and that CD26 deficiency leads to a change in the hematopoietic maturation of immune cells in the thymus (Klemann et al. 2009). However, in young animals at 1 and 3 months of age, there were no differences in leucocyte subsets, while in older animals, the T cell composition was changed significantly. Interestingly, there were no substrain-specific differences in the total number of CD4^+^ and CD8^+^ cells either in the peripheral blood or in the spleen (Klemann et al. 2009), whereas in the present study, there was a significantly higher percentage of CD4^+^ T cells in the CD26^−^ animals on dpp 7 and dpp 14. In contrast, significant differences in the total number of CD4^+^ T cells were also shown in a mouse model with CD26 knockout animals using splenic lymphocytes. Here, higher cell numbers were found in the wild types (Yan et al. 2003).

Hematopoiesis is known to be regulated by a variety of chemokines and growth factors (Broxmeyer 2008). One of them, SDF1/CXCL12, is a substrate of CD26 (Campbell and Broxmeyer 2008). The CD26 deficiency increases the homing and growth of stem cells after transplantation in the bone marrow and thus leads to an improved maturation of lymphocytes (Christopherson et al. 2004). This could explain the higher numbers of CD4^+^ T lymphocytes in the CD26^−^ animals on dpp7. Since only lung homogenates were used in the present work for the FACS analyses, a shift within the blood and lung compartments is also conceivable. It is known that CD26 is involved in the activation and transendothelial migration of T lymphocytes through its interaction with the mannose 6-phosphate/insulin-like growth factor II (Wagner et al. 2016). Different adhesion and migration of the T-lymphocytes on the vessel walls could therefore be explained by substrain-specific differences.

Interestingly, we found substrain-specific differences in the portions of activated T cells with regulatory functions characterized by CD4^+^CD25^+^ T cells during postnatal development independent of the inflammation. The population of regulatory cells comprises the CD4^+^CD25^+^ regulatory T cells (Tregs) (Demengeot et al. 2006). Their function is governed by forkhead/winged helix transcription factor foxp3 (Hori et al. 2003). We did not determine this transcription factor, so that some of the CD4^+^CD25^+^ T cells are activated CD4^+^ T cells expressing the IL2 receptor. However, previous studies showed that nearly all activated CD4^+^CD25^+^ T cells in the lung also express the Foxp3 transcription factor (Schmiedl et al. 2010). The significantly higher values of CD4^+^CD25^+^ in the CD26^−^ group in controls as well as after LPS application compared to wild types are in accordance with results using DPP4 inhibitors leading to an upregulation of regulatory T cells (Tian et al. 2010). Furthermore, earlier results exhibited a significantly increased influx of CD4^+^CD25^+^FoxP3^+^ into the lungs of CD26^−^ rats and increased IL-10-secretion/production by draining lymph node cells in culture, however, after OVA challenge (Schmiedl et al. 2010). Studies on knock-out mice showed similar results (Zhao 2019): CD26 knockout mice exhibited a delayed skin graft rejection combined with significantly reduced secretion of cytokines such as INFy, IL2, and IL4 but increased levels of IL 10 after skin transplantation. Additionally, a higher percentage of Tregs was detected in the peripheral blood lymphocytes (Zhao et al. 2019).

### LPS-induced modest parenchymal inflammation in lungs of both strains

Exposure to LPS results in an increase of activated innate immune cells, such as neutrophils and macrophages to clear the infectious agent. However, after chronic LPS exposure, only a modest tissue inflammation was visible on dpp 7 and dpp10 characterized by low numbers per area of CD11b-positive inflammatory cells in lung parenchyma, which were admittedly higher on dpp 10 than on dpp 7. However, macrophages barely increased and granulocytes increased modestly but significantly. This is in agreement with previous findings using an acute postnatal LPS inflammation model (Wagener et al. 2020) as well as with results of others (Ueda et al. 2006; Franco et al. 2002; Shrestha et al. 2019). When challenged with LPS, juvenile and adult mice show a more robust lung proinflammatory response than neonatal mice. Our data are consistent with this concept. A more pronounced LPS-induced activation of nuclear factor-light-chain enhancer in the lungs of adult compared to neonatal mice was shown (Alvira et al. 2007). Because this factor enhances B lymphocyte activation and is associated with increased lung inflammation, the airway inflammatory response was significantly greater in juvenile and adult mice than in neonatal mice (Alvira et al. 2007). Furthermore, the chemokine profile differed between juvenile and neonatal mice when challenged with LPS (Shrestha et al. 2019). In addition to this functional immaturity of the fetal immune system, the time period between LPS exposure and organ harvesting could also be important. In the adult animals, organs were obtained 9 h after LPS instillation (Zientara et al. 2019) in accordance with an established model by B. Singh, in which the inflammation peak occurs 9 h after LPS administration (Janardhan et al. 2004). Other groups reported a significant influx of inflammatory cells 6 h after LPS exposure and an inflammatory peak after about 24 h (Rinaldo et al. 1985). The extent to which these data can also be applied to newborns and young animals has not yet been clarified. In our studies, organs were harvested in the high-dose LPS group 48 h after the LPS exposure in the acute inflammatory model (Wagener et al. 2020) or during (dpp 7) and at the end (dpp10) of LPS exposure in the presented chronical LPS model. And indeed, the inflammatory reaction was at dpp 7 and 10 during chronical LPS application somewhat higher than 2 days after the end of LPS exposure (Wagener et al. 2020). So, the time of intensity of inflammation may depend on the time interval between application and investigation not only in adults but also in newborn and pups, and repeated LPS application did not result in a tolerance combined with a weakened inflammation as described by others (Collins et al. 2013; Kramer et al. 2009).

### LPS modulates the composition of leucocyte subpopulations

Within the first 10 days, the values of CD4^+^ T cells increased significantly in both groups. CD26 is expressed in different immune cells such as CD4^+^ T cells, CD8^+^ T cells, B cells, and macrophages and can additionally regulate these cells combined with the capacity to modulate a lot of cytokines and chemokines (Shao et al. 2020). Therefore, we suggested that the wild types apparently react with a stronger T-cell response to the LPS stimulus than the CD26^−^ animals and that there is a more or less pronounced upregulation of CD26^+^ T cells as seen in an asthma model of adult rats (Lun et al. 2007; Schade et al. 2008; Kruschinski et al. 2005). The missing remarkable response may be a result of the immaturity of the immune system in the neonatal mice. It is known that regulatory T cells diminish inflammatory responses by suppressing cytokine production by CD4^+^ and CD8^+^ T cells (Sakaguchi et al. 2010; Miyara and Sakaguchi 2007). Therefore, an increase of Tregs in LPS-induced animals should be expected as described for adult patients (Venet et al. 2009; Zanin-Zhorov et al. 2006; Hotchkiss et al. 2013). However, in our LPS model, the portion of CD4^+^CD25^+^ T cells decreased in both subtypes significantly independent of the developmental stage. In a chronic LPS exposure model, treating newborn mice intraperitoneally with LPS on dpp 3–5, a decrease of Tregs was also found (Shrestha et al. 2019). These findings are further supported by detecting increased airway Tregs in juvenile but not in neonatal mice after intrapharyngeal administration of LPS (McGrath-Morrow et al. 2015) and a decrease of percentage and absolute number of Tregs in fetal spleen and peripheral blood intraamniotic LPS application in a rhesus macaque model of chorioamnionitis (Rueda et al. 2016). Independent of LPS administration, Tregs decreased in the first postnatal weeks. Thus, the LPS-dependent alterations in the compositions of the immune cells are moderate and partly different to adults and may coincide with the weak inflammation seen in lung parenchyma, supporting the assumption of the functional immaturity of the perinatal immune system (Prince et al. 2004; Kramer et al. 2009; McGrath-Morrow et al. 2015).

### Influence of LPS on the lung development

A retardation of alveolarization occurred in both substrains. The total septal surface in CD26^−^ pups remained reduced compared to controls on dpp 14. However, also in controls of CD26^−^ animals, values characterizing alveolarization such as the total septal surface as well as the total septal volume were partly significantly lower and the total parenchymal airspace significantly higher. No alterations were visible in the MLI as a measure of the mean free distances in airspaces and the arithmetic mean septal thickness. Therefore, the degree of maturation of the existing alveoli is comparable in both genotypes. However, fewer new alveoli were formed. An explanation may be that CD26 is involved in tumor necrosis factor β (TGF-β) activation (Preller et al. 2007; Bauvois 2004), which is regarded as one precondition for alveolarization (Alejandre-Alcazar et al. 2008) and its interaction with fibronectin may be responsible for regular formation of septa and for timely alveolarization as discussed earlier (Wagener et al. 2020).

Thus, not only daily application of low doses of LPS for 7 days during alveolarization as carried out in this study, but also application of higher doses of LPS 1 day before and 1 day after onset of alveolarization (Wagener et al. 2020) resulted in a retardation of lung development.

Newborn rats are born with morphologically immature lungs, which are comparable with those of immature infants born at 22 to 26 weeks of gestation (Burri 2006). The most critical factors for immature infants to develop a BPD may be oxidative stress caused by high oxygen concentrations and baro/volutrauma with mechanical ventilation (Shima et al. 2013), but also prenatal and postnatal inflammation (Choi et al. 2016; Speer 2006). There are numerous rodent models of BPD using hyperoxia as inducers (D'Angio and Ryan 2014; Berger and Bhandari 2014; O'Reilly and Thebaud 2014; Schmiedl et al. 2017). Another possibility is to induce BPD-like alterations by short-term administration of LPS prenatally or postnatally as shown by some authors (Schmiedl et al. 2011; Wagener et al. 2020; Ueda et al. 2006; Hou et al. 2015; Choi et al. 2016). The aim of a daily administration of low doses of LPS over a period of 7 days (from the 3rd to 9th postnatal day) undertaken in this study was to set a chronic inflammatory stimulus. Studies on continuous postnatal LPS exposure are desirable, because there was evidence that especially postnatal infection or sepsis increased the prevalence of BPD ( Jensen and Schmidt 2014; Balany and Bhandari 2015; Choi et al. 2016).

Our chronic LPS model led to mild inflammation as described for sheep lungs (Kramer et al. 2009). Other studies indicate that lungs are able to develop some tolerance to endotoxins after repeated treatment (Kallapur et al. 2007; Kramer 2011; Gisslen et al. 2014). If these results were also applied to the early rat lung, the very first LPS exposure on day 3 would be the crucial inflammatory stimulus, which would have subsided on days 7 and 10. Choi and colleagues (Choi et al. 2009) demonstrated that LPS administered during the saccular period interrupts alveolarization and pulmonary vascularization at dpp 7 and dpp 14.

## Conclusions

Chronical application of LPS leads to a weak inflammatory response, and to a retardation of pulmonary development also seen in animal models using higher dosed LPS.

Looking at the immune status, CD26-dependent differences are already seen without LPS, confirming partly the mediator role of CD26 during T cell activation. Chronical LPS exposure results in moderate CD26-dependent alterations in the immune cell contents, leading in CD26^−^ pups to a retarded increase of CD4 ^+^ T cells combined with a significant increase of monocytes/macrophages.
